# Significance of NLDA, the commixed index of inflammation, immune responses, hemostasis, and nutrition, for predicting metastatic non-small cell lung cancer prognosis and metastases

**DOI:** 10.18632/oncotarget.18184

**Published:** 2017-05-23

**Authors:** Wen-wen Sun, Jia-an Hu, Wen-quan Niu, Bei-li Gao, Zhi-hong Xu

**Affiliations:** ^1^ Department of Geriatrics, Ruijin Hospital, Shanghai Jiaotong University School of Medicine, Shanghai 200025, People’s Republic of China; ^2^ Department of Respiration, Ruijin Hospital, Shanghai Jiaotong University School of Medicine, Shanghai 200025, People’s Republic of China; ^3^ State Key Laboratory of Medical Genomics, Ruijin Hospital, Shanghai Jiaotong University School of Medicine, Shanghai 200025, People’s Republic of China

**Keywords:** NLDA, inflammation-immune-hemostasis-nutrition indexes, predictive prognostic model, organ metastases, non-small cell lung cancer

## Abstract

**Purpose:**

This study aimed to take a comprehensive review of the hematological indexes and discover a novel, comprehensive, and economical index for prognostic prediction.

**Results:**

The predictive prognostic model revealed that an elevated value of NLDA (NLDA = neutrophil count/lymphocyte count × D-dimer count/albumin) was an independent risk factor for one-year adverse prognosis (hazard ratio = 3.038; 95% confidence interval [CI], 1.959–4.712; *P* < 0.001). The C-indexes of internal and external validation in nomogram were 0.738 (95% CI, 0.686–0.79) and 0.731 (95% CI, 0.631–0.831), respectively. The areas under the curves of the NLDA values in retrospective and prospective studies were 0.700 (95% CI, 0.631–0.769; *P* < 0.001) and 0.692 (95% CI, 0.535–0.822; *P* = 0.005), respectively. The cut-off value of NLDA was 0.15. NLDA was positively associated with M stage (*P* = 0.032), organ metastasis counts (*P* = 0.006), liver metastases (*P* = 0.019), and vertebrae metastases (*P* = 0.013).

**Materials and Methods:**

This was a retrospective and prospective study. The clinicopathological characteristics and hematological parameters of stage IV non-small cell lung cancer patients were analyzed retrospectively and prospectively to establish a valid predictive prognostic model. The primary endpoint was the 1-year overall survival. The predictive prognostic model was established and validated by Cox Regression and nomogram. The cut-off and predictive prognostic values of the novel indexes were calculated through the receiver operating characteristic curves. The chi-square test was used to explore the correlation between the new prognostic hematological index and metastatic characteristics.

**Conclusions:**

In this study, NLDA, a new, comprehensive and economic parameter, was found to be an independent adverse prognostic factor for stage IV non-small cell lung cancer patients, and was positively associated with organ metastases.

## INTRODUCTION

Data from the Globocan 2012 show that the high incidence and mortality rates of lung cancer have made it the deadliest type of cancer except for melanocytoma [1]. The biological behavior of tumors can no longer be understood simply by calculating the traits of the cancer cells but instead must covering the contributions of the “tumor microenvironment” [2]. The Risk prediction models for lung cancer typically include age, gender, cigarette smoking duration, medical history, tumor stage and pathological type; however, prognostic model that includes the comprehensive indexes of tumor microenvironment is scarcely taken into consideration in lung cancer patients. To get a better understanding of the tumor microenvironment for the prognosis of lung cancer patients, a validated model is needed to better define the poor prognostic factors.

Experimental studies have testified that the tumor microenvironment concerning the inflammatory, immune, hemostasis-coagulation, nutrition metabolism plays crucial role in the formation and progression of the malignancies by promoting DNA damage, tumor cell proliferation, angiogenesis, and metastasis and reducing apoptosis and anti-cancer agent responses [3–5]. Some clinical researches showed that hematological indexes of systemic hemostasis-coagulation and inflammatory were negatively associated with prognosis, but indexes of systemic immune were positively associated with prognosis in various cancers [6–14]. Other hematological indexes regarding nutrition status such as hemoglobin and albumin have also been reported as potential predictors of survival [15–17].

However, the prognostic significance of the comprehensive hematological indexes containing the above four aspects is scarcely studied in clinic. Besides, the current hematological tumor-related indexes do not possess high specificity for predicting prognosis. Other diagnostic methods with higher accuracy, such as circulating tumor cells (CTCs) and circulating DNA, are too expensive for patients with lung cancer. The objective of this study was to take a comprehensive review of the hematological indexes and discover a novel, comprehensive, and economic index for predicting prognosis.

## RESULTS

### Baseline characteristics of the patients in the retrospective study

Of 785 patients diagnosed with lung cancer, 272 stage IV NSCLC patients met the inclusion criteria and were enrolled in the retrospective study group. In this study, there were 95 non-survivors, 144 survivors, and 33 patients who were lost to follow-up. The death rates of male and currently smoking patients were significantly higher than those of female and non-currently smoking patients. Besides, the survival time of male and currently smoking patients was generally shorter than that of female and non-currently smoking patients (*P* = 0.036 and 0.002, respectively). Significantly higher death rates were observed with the elevation of N stage, M stage, and the increase of organ metastases counts (*P* = 0.009, 0.029, and 0.005, respectively). However, there were no statistical differences in death rate in terms of age, tumor location pathological type, TNM stage, T stage, therapy type, or CEA level. A detailed analysis of the 272 patients is shown in Table 1.

**Table 1:** The clinicopathological characteristics of the retrospective study subgroup

	Censored patients *n* (%)	Nonsurvivors *n* (%)	Median survival time (interquartile range)	*P*
Age				
< 65	105 (38.6)	54 (19.9)	365 (192, 365)	0.692
≥ 65	72 (26.5)	41 (15.1)	335 (124, 365)	
Gender				
Male	98 (36)	65 (23.9)	313 (141, 365)	0.036
Female	79 (29)	30 (11)	365 (214, 365)	
Current smoking				
No	102 (37.5)	36 (13.2)	365 (244, 365)	0.002
Yes	75 (27.6)	59 (21.7)	286.5 (112.5, 365)	
PS				
0–1	143 (52.6)	68 (25)	365 (192, 365)	0.082
2	34 (12.5)	27 (9.9)	294 (95.5, 365)	
Tumor location				
Right lung	96 (35.3)	61 (22.4)	365 (162, 365)	0.112
Left lung	81 (29.8)	34 (12.5)	365 (172, 365)	
Pathological type				
Squamous carcinoma	34 (12.5)	16 (5.9)	349 (135.5, 365)	0.631
Adenocarcinoma and adenosquamous carcuinoma	143 (52.6)	79 (29)	365 (170.5, 365)	
TNM Stage				
IVa	67 (24.6)	25 (9.2)	365 (193.75, 365)	0.055
IVb	110 (40.4)	70 (25.7)	336 (146.75, 365)	
T stage				
T1–T2	41 (15.1)	17 (6.3)	365 (201.25, 365)	0.312
T3–T4	136 (50)	78 (28.7)	365 (157.75, 365)	
N stage				
N0–N2	85 (31.3)	30 (11)	365 (212, 365)	0.009
N3	92 (33.8)	65 (23.9)	313 (154.5, 365)	
M stage				
1a	51 (18.8)	16 (5.9)	365 (233, 365)	0.029
1b–1c	126 (46.3)	79 (29)	335 (124, 365)	
Organ metastasis counts				
< 2	69 (25.4)	21 (7.7)	365 (227.5, 365)	0.005
≥ 2	108 (39.7)	74 (27.2)	326 (144.25, 365)	
Therapy				
Chemotherapy	141 (51.8)	84 (30.9)	365 (164, 365)	0.068
Targeted drugs	36 (13.2)	11 (4.0)	365 (163, 365)	
CEA				
< 5 ng/ml	70 (25.7)	45 (16.5)	314 (125, 365)	0.213
≥ 5 ng/ml	107 (39.3)	50 (18.4)	365 (211.5, 365)	

Abbreviations: PS, Performance Status; CEA, carcinoembryonic antigen.

### Inflammation-immune-hemostasis-nutrition indexes in the retrospective study

In the analysis of the inflammatory-immune indexes, the subgroup of NLR > 3 had a statistically higher incidence of death than the subgroup of NLR ≤ 3 (*P* < 0.001). The death rates of the patients with a D-dimer > 0.55 mg/L, Fg ≥ 3.5 g/L, FDP ≥ 5 mg/L were 27.6%, 22.8%, and 17.3%, respectively, which were significantly higher than those of the patients with normal levels of the hemostasis indexes, and all *P* values were < 0.001. A significant difference of the death rate was observed between the patients with albumin level ≥ 35 g/L and those with albumin level < 35 g/L, but no statistically significant differences were seen in terms of other nutrition indexes. The death rates of the subpopulations with PDM > 272, SII ≥ 870, PNI < 45, NLFDPA > 0.54, NLFgA > 0.92, and NLDA > 0.15 were all were significantly higher than the opposite subgroups respectively (*P* < 0.001 for all). The median survival time of the abnormal subpopulations was generally shorter than that of the normal subpopulations in terms of the different independent factors (Table 2).

**Table 2:** The inflammation-immune-hemostasis-nutrition hematological indexes of the retrospective study subgroup

	Censored patients *n* (%)	Nonsurvivors *n* (%)	Median survival time (interquartile range)	*P*
1. Inflammation-immune based hematological indexes				
WBC				
≤ ULN	141 (51.8)	72 (26.5)	365 (170.5, 365)	0.46
> ULN	36 (13.2)	23 (8.5)	345 (111, 365)	
Neutrophil				
≤ 7*10^9/L	151 (55.5)	77 (28.3)	365 (169.75, 365)	0.363
> 7*10^9/L	26 (9.6)	18 (6.6)	339 (105.5, 365	
Lymphocyte				
≥ 0.8*10^9/L	171 (62.9)	91 (33.5)	365 (157.75, 365)	0.732
< 0.8*10^9/L	6 (2.2)	4 (1.5)	304 (222.25, 365)	
NLR				
< 3	83 (30.5)	23 (8.5)	365 (225.5, 365)	< 0.001
≥ 3	94 (34.6)	72 (26.5)	306 (140.5, 365)	
NLMR				
≤ 3.4	152 (55.9)	74 (27.2)	365 (179.75, 365)	0.094
> 3.4	25 (9.2)	21 (7.7)	324.5 (124.25, 365)	
2. Hemostasis based hematological indexes				
Platelet				
≤ ULN	144 (52.9)	73 (26.8)	365 (170.5, 365)	0.377
> ULN	33 (12.1)	22 (8.1)	335 (141, 365)	
D-dimer				
≤ 0.55 mg/L	91 (33.5)	20 (7.4)	365 (303, 365)	< 0.001
> 0.55 mg/L	86 (31.6)	75 (27.6)	283 (117.5, 365)	
Fg				
≤ 3.5 g/L	102 (37.5)	33 (12.1)	365 (221, 365)	< 0.001
> 3.5 g/L	75 (27.6)	62 (22.8)	294 (133, 365)	
FDP				
≤ 5 mg/L	139 (51.1)	48 (17.6)	365 (240, 365)	< 0.001
> 5 mg/L	38 (14)	47 (17.3)	226 (84, 365)	
3. Nutrition based hematological indexes				
RBC				
≥ LLN	141 (51.8)	71 (26.1)	365 (169.75, 365)	0.35
< LLN	36 (13.2)	24 (8.8)	365 (115.5, 365)	
Hemoglobin				
≥ LLN	123 (45.2)	59 (21.7)	365 (191.25, 365)	0.217
< LLN	54 (19.9)	36 (13.2)	324.5 (110.25, 365)	
Albumin				
≥ 35 g/L	100 (36.8)	27 (9.9)	365 (245, 365)	< 0.001
< 35 g/L	77 (28.3)	68 (25	292 (127.5, 365)	
4. Commixed hematological indexes				
PLR				
< 200	141 (51.8)	66 (24.3)	365 (166, 365)	0.06
≥ 200	36 (13.2)	29 (10.7)	303 (150, 365)	
PDM				
≤ 272	140 (51.5)	41 (15.1)	365 (233, 365)	< 0.001
> 272	37 (13.6)	54 (19.9)	226 (83, 365)	
SII				
< 870	123 (45.2)	43 (15.8)	365 (223.25, 365)	< 0.001
≥ 870	54 (19.9)	52 (19.1)	246.5 (96, 365)	
PNI				
≥ 45	84 (30.9)	17 (6.3)	365 (277.5, 365)	< 0.001
< 45	93 (34.2)	78 (28.7)	297 (130, 365)	
NLFDPA				
≤ 0.54	142 (52.2)	43 (15.8)	365 (244, 365)	< 0.001
> 0.54	35 (12.9)	52 (19.1)	211 (82, 365)	
NLFgA				
≤ 0.92	167 (61.4)	81 (29.8)	365 (172.25, 365)	0.012
> 0.92	10 (3.7)	14 (5.1)	233 (105.5, 365)	
NLDA				
≤ 0.15	149 (54.8)	42 (15.4)	365 (241, 365)	< 0.001
> 0.15	28 (10.3)	53 (19.5)	192 (82.5, 365)	

Abbreviations: WBC, white blood cell; ULN, upper limit of normal; NLR = N/L; NLMR = N/(L+M); Fg, fibrinogen; FDP, fibrin degradation product; RBC, red blood cells; LLN, lower limit of normal; PLR = P/L; PDM = P × D; SII = P × N/L; PNI = albumin + 5 × L; NLFDPA = N × FDP/(L × albumin); NLFgA = N × Fg/(L × albumin); NLDA = N × D/(L × albumin).

### Prognostic significance of the inflammation-immune-hemostasis-nutrition indexes in the retrospective study

**Figure 1:**
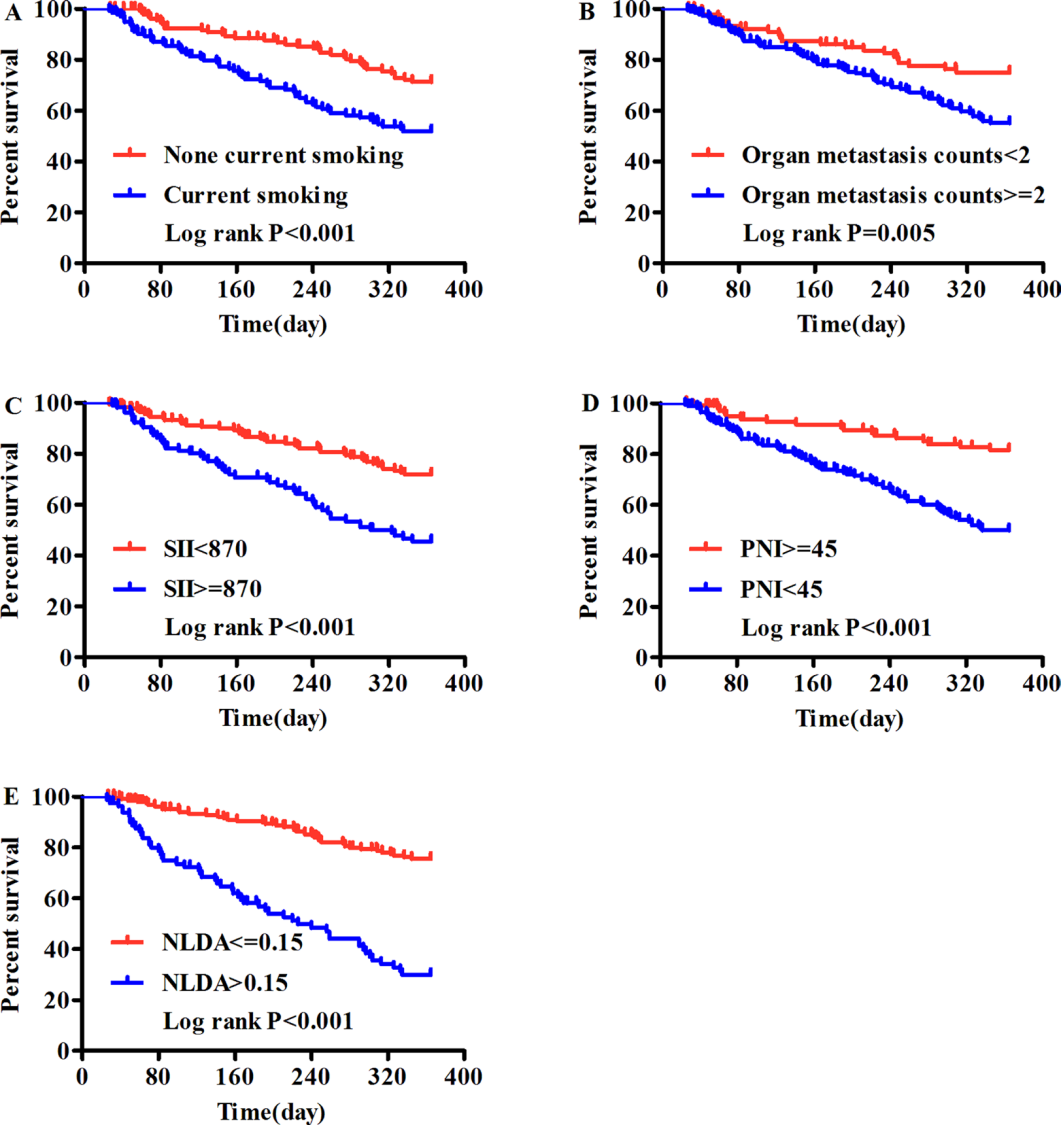
The 1-year overall survival analysis of IV stage NSCLC patients using Kaplan - Meier survival method (**A**) Current smoking, (**B**) Organ metastasis counts, (**C**) SII, (**D**) PNI and (**E**) NLDA which were identified to be the five independent prognostic factors by multivariate survival analysis.

The Univariate Cox Regression analysis showed that in addition to gender, current smoking status, (PS) , N stage, M stage and organ metastases counts, abnormal nutrition-based hematological indexes (albumin), inflammatory-immune based hematological indexes (NLR and NLMR), hemostasis-based hematological indexes (D-dimer, Fg, FDP and PDM), and commixed hematological indexes (SII, PNI, NLFDPA, NLFgA, and NLDA) were negatively associated with the prognosis in patients with NSCLC. However, in the multivariate Cox Regression analysis, only five factors independently predicted poor prognosis: current smoking (HR, 1.774; 95% CI, 1.153–2.731; *P* = 0.009), organ metastasis counts (HR, 1.732; 95% CI, 1.045–2.870; *P* = 0.033), SII (HR, 1.809; 95% CI, 1.178–2.776; *P* = 0.007), PNI (HR, 2.311; 95% CI, 1.297–4.118; *P* = 0.010) and NLDA (HR, 3.038; 95% CI, 1.959–4.712; *P* < 0.001) (Table 3). The HR of each variable in multivariate Cox Regression has been adjusted. These results were also confirmed by Kaplan-Meier analysis using log-rank methods (Figure 1).

**Table 3:** Univariate and multivariate Cox regression analysis of the clinicopathological characteristics and hematological indexes

	Univariate Cox regression HR (95%CI)	*P*	Multivariate Cox regression HR (95%CI)	*P*
Age	1.161 (0.774, 1.743)	0.47	-	-
Gender	0.606 (0.393, 0.934)	0.023	-	-
Current smoking	2.067 (1.365, 3.131)	0.001	1.774* (1.153, 2.731)	0.009
PS	1.578 (1.010, 2.465)	0.045	-	-
Tumor location	0.714 (0.469, 1.087)	0.116	-	-
Pathological type	1.049 (0.613, 1.795)	0.862	-	-
TNM Stage	1.544 (0.978, 2.439)	0.062	-	-
T stage	1.308 (0.774, 2.210)	0.316	-	-
N stage	1.779 (1.154, 2.743)	0.009	-	-
M stage	1.792 (1.047, 3.068)	0.033	-	-
Organ metastasis counts	1.977 (1.217, 3.210)	0.006	1.732* (1.045, 2.870)	0.033
Therapy	0.602 (0.321, 1.129)	0.114	-	-
CEA	0.717 (0.479, 1.073)	0.106	-	-
WBC	1.228 (0.768, 1.963)	0.392	-	-
Neutrophil	1.284 (0.769, 2.146)	0.339	-	-
Lymphocyte	1.065 (0.391, 2.9)	0.902	-	-
NLR	2.308 (1.443, 3.694)	< 0.0001	-	-
NLMR	1.521 (0.937, 2.47)	0.09	-	-
Platelet	1.244 (0.773, 2.005)	0.369	-	-
D-dimer	3.326 (2.028, 5.453)	< 0.0001	-	-
Fg	2.15 (1.408, 3.282)	< 0.0001	-	-
FDP	2.949 (1.968, 4.420)	< 0.0001	-	-
RBC	1.236 (0.778, 1.963)	0.37	-	-
Hemoglobin	1.383 (0.913, 2.93)	0.126	-	-
Albumin	2.67 (1.708, 4.174)	< 0.0001	-	-
PLR	1.462 (0.945, 2.264)	0.088	-	-
PDM	3.605 (2.397, 5.423)	< 0.0001	-	-
SII	2.404 (1.603, 3.606)	< 0.0001	1.809* (1.178, 2.776)	0.007
PNI	3.328 (1.968, 5.628)	< 0.0001	2.322* (1.297, 4.118)	0.010
NLFDPA	3.759 (2.501, 5.65)	< 0.0001	-	-
NLFgA	2.167 (1.228, 3.826)	0.008	-	-
NLDA	1.701 (1.424, 2.032)	< 0.0001	3.038* (1.959, 4.712)	< 0.0001

Abbreviations: PS, Performance Status; CEA, carcinoembryonic antigen; WBC, white blood cell; ULN, upper limit of normal; NLR = N/L; NLMR = N/(L+M); Fg, fibrinogen; FDP, fibrin degradation product; RBC, red blood cells; LLN, lower limit of normal; PLR = P/L; PDM = P × D; SII = P × N/L; PNI = albumin + 5 × L; NLFDPA = N × FDP/(L × albumin); NLFgA = N × Fg/(L × albumin); NLDA = N × D/(L × albumin). The linear correlation test has been applied to investigate cox proportional hazards assumption. The variables, occupying statistical significance in Univariate Cox Regression, were analyzed in Multivariate Cox Regression. The interaction terms between those covariates were taken into consideration. * HR had been adjusted.

### Nomogram and internal validation of the predictive prognostic model in the retrospective study

The nomogram was applied to further verify the predictive prognostic model of 1-year overall survival for stage IV NSCLC patients. The hematological indexes including current smoking history, organ metastases counts, SII, PNI, and NLDA which were analyzed out by Univariate and multivariate Cox Regression in the retrospective study were taken into the nomogram program. The result of the nomogram were in accordance with that of the multivariate survival analyses, and NLDA was identified as the poorest prognostic factor in this retrospective study.

**Figure 2:**
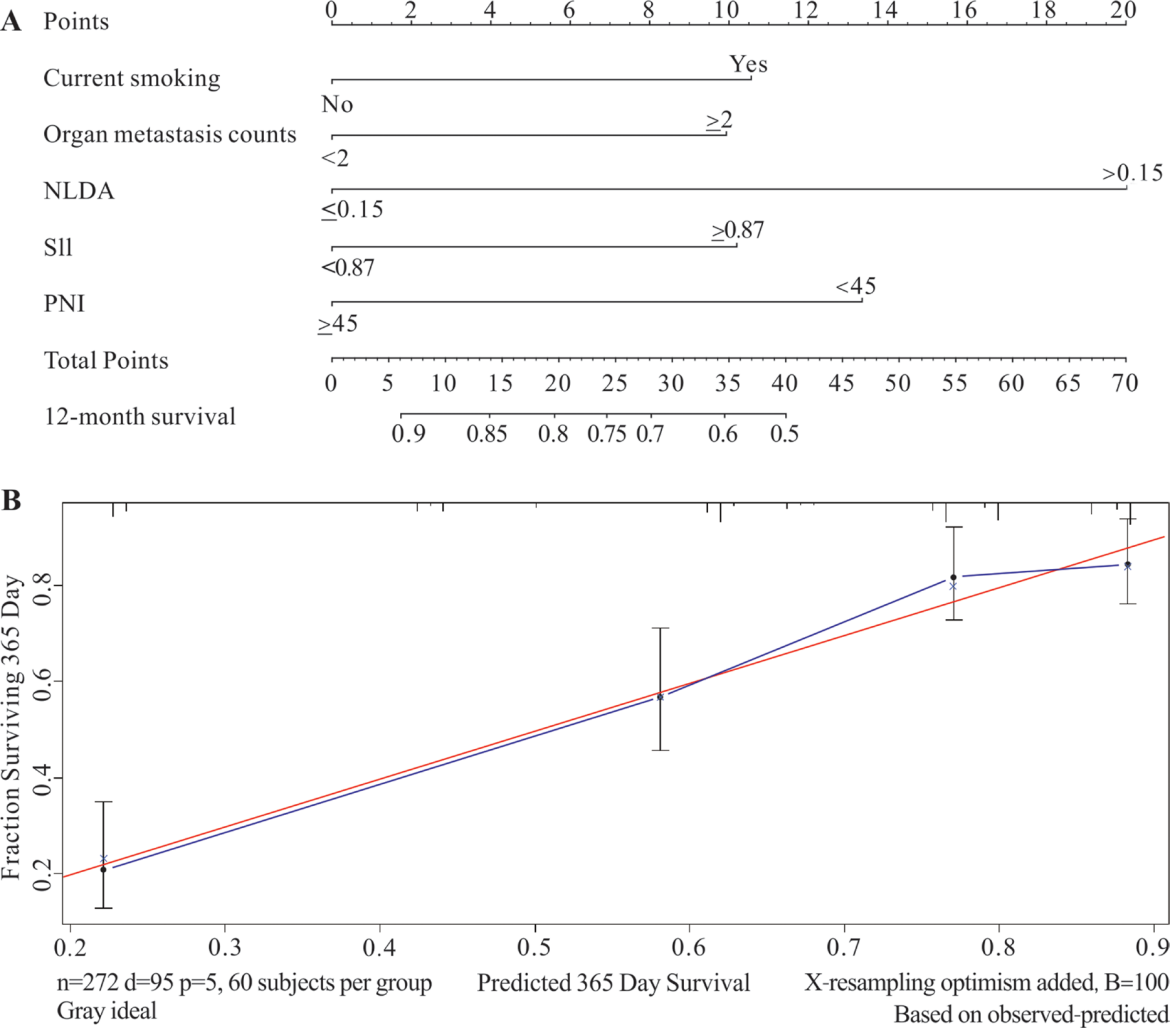
The nomogram and calibration curve of the retrospective study group The retrospective study was applied to establish the predictive prognostic model and to conduct internal validation. (**A**) The predictive prognostic model which illustrated that the value of NLDA was identified as the poorest prognostic factor in this study group. (**B**) The C-index of the internal validation was 0.738 (95% CI, 0.686–0.79) and the calibrated curve showed that the predictive model had high calibration with the clinic.

The consequences of the internal validation depend on the parameter of C-index and calibration curve of the retrospective study. The C-index of the prodictive prognostic model was 0.738 (95% CI, 0.686–0.79) and the C-index of the TNM staging system was 0.576 (95% CI, 0.516–0.634), which implied that the predictive prognostic accuracy of this model was significantly better than that of the TNM staging system. The result of the calibration curve demonstrated that the predictive probability of 1-year survival was close to the actual 1-year survival in clinic (Figure 2).

### External validation of the predictive prognostic model in the prospective study

**Figure 3:**
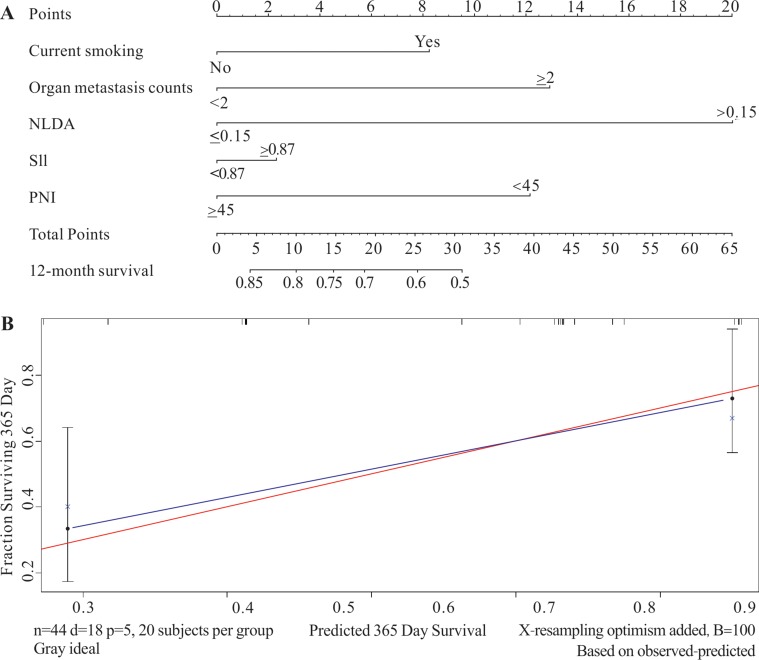
The nomogram and calibration curve of the prospective study group The prospective study was used as external validation. (**A**) In the prospective study, the value of NLDA was also identified as the poorest prognostic factor in this study group. (**B**) The C-index was 0.731 (95% CI, 0.631–0.831) and the calibrated curve testified that the predictive model which was applied in the prospective study had high calibration with the clinic as well.

A total of 44 subjects (18 non-survivors, 22 survivors, and four lost to follow-up) were recruited in the prospective study group according to the inclusion and exclusion criteria which were extremely similar to those of the retrospective population. The basic clinical characteristics and five independent prognostic factors which were analyzed in prospective study were displaied in Table 4. The five factors were analyzed collectively in the nomogram, and NLDA was still the poorest independent prognostic factor (Figure 3). The C-index was 0.731 (95% CI, 0.631–0.831) and the calibrated curve testified that the predictive model had high calibration with clinical (Figure 3). The results of the external validation were generally consistent with the internal validation; hence, through the analysis of the prospective study, the predictive prognostic model was identified to be comparatively accurate.

**Table 4:** The clinicopathological characteristics of the prospective study subgroup

	Censored Patients *n* (%)	Nonsurvivors *n* (%)	Median Survival time (interquartile range)	*P*
Age				
< 65	14 (31.8)	10 (22.7)	309 (94, 365)	0.911
≥ 65	12 (27.3)	8 (18.2)	365 (183.25, 365)	
Gender				
Male	18 (40.9)	14 (31.8)	282 (94, 365)	0.531
Female	8 (18.2)	4 (9.1)	365 (232.5, 365)	
Current smoking				
No	11 (25)	5 (11.4)	365 (264, 365)	0.325
Yes	15 (34.1)	13 (29.5)	192 (88.5, 365)	
PS				
0–1	21 (47.7)	12 (27.3)	365 (123, 365)	0.288
2	5 (11.4)	6 (13.6)	217 (87, 365)	
Tumor location				
Right lung	12 (27.3)	13 (29.5)	279 (123, 365)	0.086
Left lung	14 (31.8)	5 (11.4)	365 (97, 365)	
Pathological type				
Squamous	8 (18.2)	8 (18.2)	251 (79, 365)	0.354
Adenocarcinoma and adenosquamous carcinoma	18 (40.9)	10 (22.7)	365 (145.25, 365)	
TNM Stage				
IVa	16 (36.4)	7 (15.9)	365 (97, 365)	0.139
IVb	10 (22.7)	11 (25)	217 (123, 365)	
Organ metastasis counts				
< 2	17 (38.6)	5 (11.4)	365 (218.5, 365)	0.014
≥ 2	9 (20.5)	13 (29.5)	195 (99, 365)	
Therapy				
Chemotherapy	21 (47.7)	14 (31.8)	312 (97, 365)	0.809
Targeted drugs	5 (11.4)	4 (9.1)	365 (160, 365)	
PNI				
≥ 45	13 (29.5)	5 (11.4)	365 (173.5, 365)	0.135
< 45	13 (29.5)	13 (29.5)	238 (91.5, 365)	
SII				
< 870	20 (45.5)	10 (22.7)	365 (96, 365)	0.135
≥ 870	6 (13.6)	8 (18.2)	309 (183.5, 365	
NLDA				
≤ 0.15	23 (52.3)	9 (20.5)	365 (189.5, 365)	0.005
> 0.15	3 (6.8)	9 (20.5)	147.5 (69.75, 283.75)	

Abbreviations: PS, Performance Status; SII = P × N/L; PNI = albumin + 5 × L; NLDA = N × D/(L × albumin).

### Predictive values of the novel NLDA hematological index

**Figure 4:**
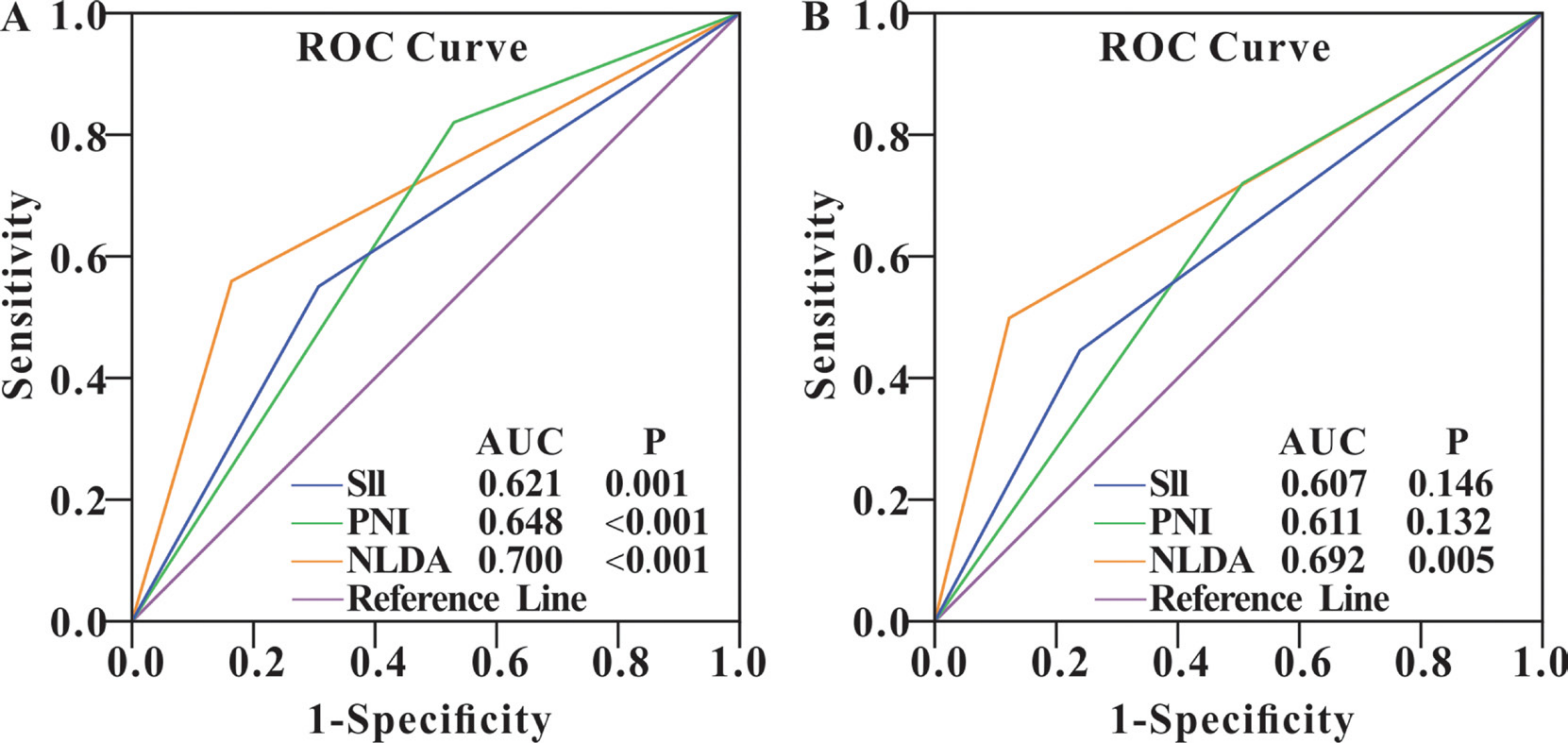
ROC curves of SII, PNI and NLDA in retrospective and prospective study group The results of the retrospective study group (**A**) and the prospective study group (**B**) showed that the elevation of NLDA level was more significantly and accurately predicted 1-year overall survival of the NSCLC patients than other hematological indexes in this whole study.

To evaluate the predictive accuracy of the NLDA index for overall survival, we conducted the ROC curves analysis of NLDA and other indexes including the NLR, PDM, SII, and PNI in the retrospective study and prospective study respectively. The results of both of the studies showed that compared with other hematological indexes an elevated NLDA could predict the NSCLC patients’ prognosis of 1-year overall survival more accurately (Figure 4). In the retrospective set, NLDA possessed a AUC of 0.700 (95% CI, 0.631–0.769; *P* < 0.001), the specificity of 84.18% and sensitivity of 55.79%; in the prospective set, the AUC of the set was 0.692 (95% CI, 0.535–0.822; *P* = 0.005), the sensitivity and specificity of NLDA were 50% and 88.46%, respectively (Table 5). Besides, the cut-off NLDA value was 0.15 calculated out by the MedCalc software.

**Table 5:** The predictive value of NLDA for NSCLC 1-year overall survival

	Sensitivity (95% CI)	Specificity 95% CI	AUC (95% CI)	*P*
Retrospective study subgroup	55.79 (45.2–66.0)	84.18 (78.0–89.2)	0.7 (0.631–0.769)	< 0.001
Prospective study subgroup	50 (26.0–74.0)	88.46 (69.8–97.6)	0.692 (0.535–0.822)	0.005

Abbreviations: NLDA, NLDA = N × D/(L × albumin); NSCLC, non-small cell lung cancer; AUC, area under curve.

### Relationship between NLDA and organ metastases

**Figure 5:**
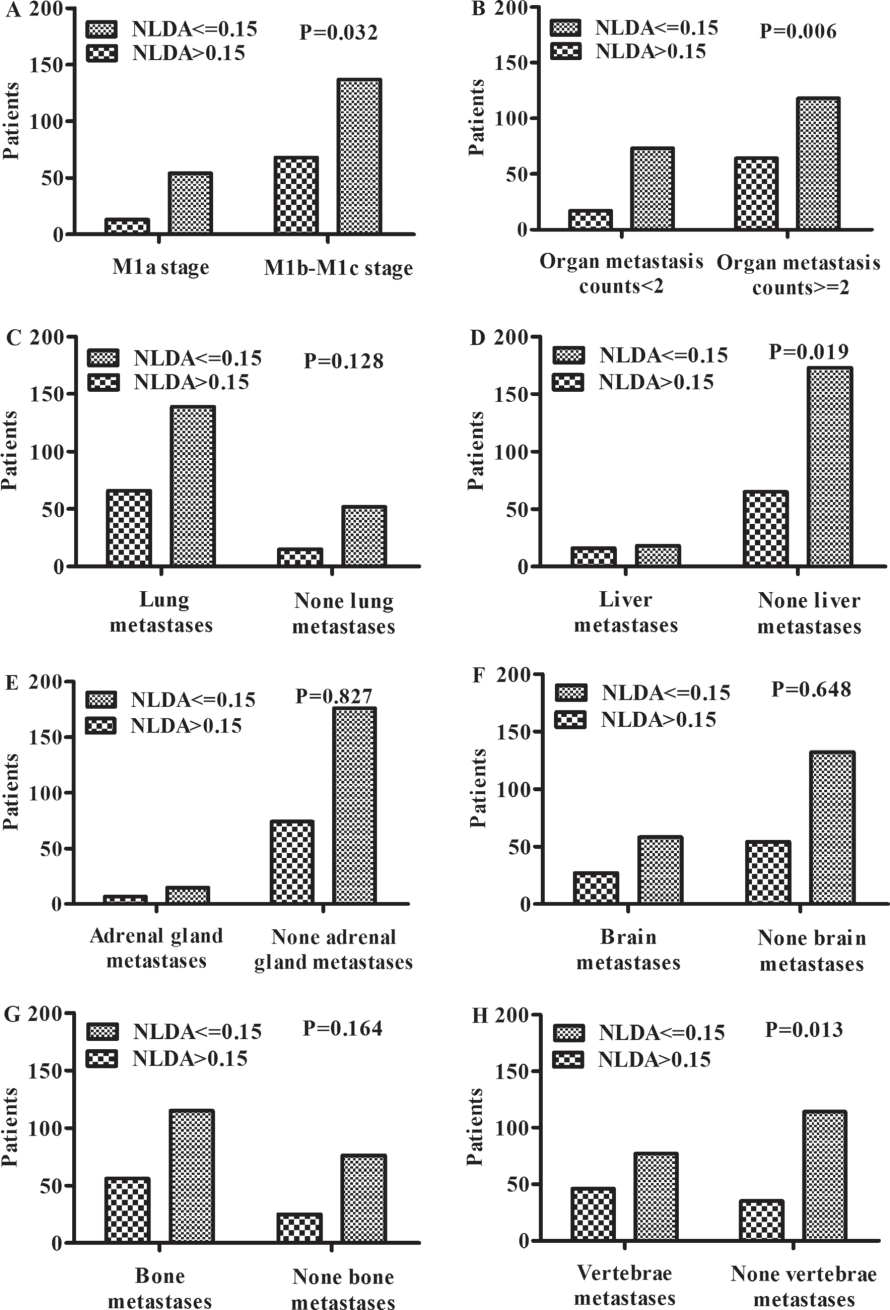
NLDA and organ metastases The distribution of NLDA differed significantly in (**A**) M stage (*P* = 0.032), (**B**) organ metastasis counts (*P* = 0.006), (**D**) liver metastases (*P* = 0.019) and (**H**) vertebrae metastases (*P* = 0.013), but this study did not show that (**C**) lung metastases, (**E**) adrenal gland metastases, (**F**) brain metastases, and (**G**) bone metastases have association with NLDA.

The chi-square test analysis showed that the distribution of NLDA > 0.15 was significantly higher in M1b-M1c stage (*P* = 0.032), organ metastasis counts > = 2 (*P* = 0.006), vertebrae metastases (*P* = 0.013), and liver metastases (*P* = 0.019), but did not show significant elevation in lung metastases, bone metastases, brain metastases, and adrenal gland metastases (Figure 5). Our findings indicated that the occurrence of metastasis have positive association with the level of NLDA and the level of NLDA could reflect the progression of NSCLC.

## DISCUSSION

This study found that the death rate was generally higher in the subgroup with abnormal hematological indexes than that in the subgroup with normal hematological indexes, which was in accordance with many other studies [6–14]. NLDA, a novel index, was identified as the independent adverse factor in the prognostic prediction of NSCLC by internal and external validation. NLDA which was established on the pretreatment values of neutrophils, lymphocytes, D-dimer, and albumin represented the four different characteristics of the tumor microenvironment.

In addition to NLDA, the hematological indexes of NLR, NLMR, D-dimer, Fg, FDP, PDM, SII, PNI, NLFDPA, NLFgA, and NLDA were also found to be significantly associated with 1-year overall survival in the univariate survival analysis, whereas only the indexes of SII, PNI, and NLDA were identified to be the independent adverse prognostic factors in multivariate Cox Regression analysis. Some reports have also found that the abnomalities of SII and PNI levels were related to metastasis and considered them valuable predictive indicators of cancer prognosis [18–21]. The number of organ metastases was included in the multivariate Cox Regression analysis rather than the clinical TNM stage. The reasons may be that the all the patients belong to clinical stage IV and organ metastases count is the specification of clinical TNM stages.

The nomogram was established to show the impact of hematological parameters on the prognosis of patients with NSCLC. NLDA was included in the nomograms of the retrospective study and prospective study. Moreover, both of the two studies compared the predictive accuracies of the novel predictive prognostic model and TNM stage system. The results showed that the accuracy of the nomogram with NLDA and other parameters was better than that of TNM stage system.

We also found that NLDA was significantly associated with M stage, organ metastasis counts, vertebrae metastases, and liver metastases. Metastases are considered the prime cause of death in patients with lung cancer [22]. The bone was identified to be the most frequent homing site for circulating tumor cells and the most common reservoir for disseminated tumor cells [23, 24]. The NLDA index was shown to have an association with vertebrate metastases. The occurrence and progression of metastases has a tight correlation with the tumor microenvironment, including inflammation, immune, hemostasis, and nutrition [4, 5]. Since there was a close relationship between metastases and NLDA, NLDA can also be considered an index reflecting organ metastases.

All the above hematological indexes belong to the categories of inflammation, immunity, hemostasis, and nutrition; hence, a better understanding of the role of neutrophils, lymphocytes, D-dimer, and albumin in cancer would help us clarify the associations between cancer and inflammation, immunity, hemostasis, and nutrition. The following discussions mainly focus on the four properties.

Tumors are often infiltrated by various numbers of lymphocytes, neutrophils, macrophages, and mast cells. Sakurai et al. [25] assumed that tumor-promoting inflammation and antitumor immunity coexist at different points during the period of tumor progression and that the environmental and microenvironmental conditions dictated the balance between the two. Tumor-associated macrophages (TAMs) and T cells are the most frequently found immune cells within the tumor microenvironment [26].

As to the inflammatory, experimental evidences showed that TAMs mostly contribute to tumor growth and may be responsible for angiogenesis, invasion, and metastasis, and in addition to the inflammatory cells, the cytokine and chemokine may have more relevant to enhance cancer cell proliferation, invasion, and metastasis by evading immune surveillance, promoting angiogenesis, and facilitating genomic instability [26–30]. T-cell activation involves both stimulatory and inhibitory checkpoint signals while increased T cell counts, specifically activated cytotoxic lymphocytes (CTLs) and T-helper 1 (Th1) cells, correlate with better survival in some cancers, including invasive colon cancer, melanoma, multiple myeloma, and pancreatic cancer [31, 32]. Our study also revealed that, although the combined indexes of NLR and NLMR were not identified as independent prognostic factors, they were proven to have an association with survival. Some studies also showed that NLR was related to poor prognosis of patients with NSCLC as well as those with advanced hepatocellular carcinoma and gastric cancer [7–9].

D-dimer composed the NLDA index and was identified to be correlated with survival. Compared with FDP and cross-linking fibrin, D-dimer is a more stable end product of fibrin degradation that is deposited in and around solid tumors [3]. In addition to NLDA and D-dimer, our study found that Fg , FDP, and PDM showed significance in the univariate survival analysis. Palumbo et al. [33] found that the most important action of fibrinogen was facilitating the circulating tumor cells to form spontaneous metastases. Many other clinical studies demonstrated that activation of the hemostatic system and the extent of this activation were associated with a more advanced tumor stage, unfavorable outcome, and patient prognosis in a variety of solid tumors [10–13]. The mechanism are presented as below. As tumor cells are released from the primary tumor into the circulation, platelet–fibrin clots deposite around the tumor cells. These clots may protect tumor cells from innate immune surveillance systems [33–34]. Besides, the platelet could produce platelet-derived endothelial cell growth factor [35].

Third, as a chronic consumption disease, NSCLC may lead to albumin catabolism during cancer progression as well as nutrition absorption disorders. These two causes might induce a decrease in the level of albumin. Some previous studies showed that hypoalbuminemia was associated with poor prognosis [36]. Lower serum albumin can reflect tumor-bearing conditions with systemic inflammation [37]. Several studies have found that the deterioration of hypoalbuminemia is secondary to the elevation of serum CRP, as many cancer patients with hypoalbuminemia already have increased serum CRP levels [38]. Besides, Chen at al. [39] found that hemoglobin was a significant prognostic factor.

However, the sensitivity of NLDA was low, the reasons might be that the follow-up time was comparatively brief and the sample size of the two study groups, especially the prospective group, were small. Longer time follow-up and larger sample size clinical researches should be conducted. Moreover, in the clinical setting, some patients with NSCLC always have mild or moderate obstructive pneumonia caused by the tumor, particularly in cases of central lung carcinoma, or the tumor lesions are mistaken for inflammatory lesions; therefore, whether NLDA is applicable in such cases requires further clinical study. In addition, although a laboratory study showed that anti-inflammatory therapy effectively prevented early neoplastic progression and malignant conversion [27], whether the anti-inflammatory therapy could aid the prognosis of patients with NSCLC remains to be elucidated. To our knowledge, no study to date has examined this issue.

## MATERIALS AND METHODS

### Study design

**Figure 6:**
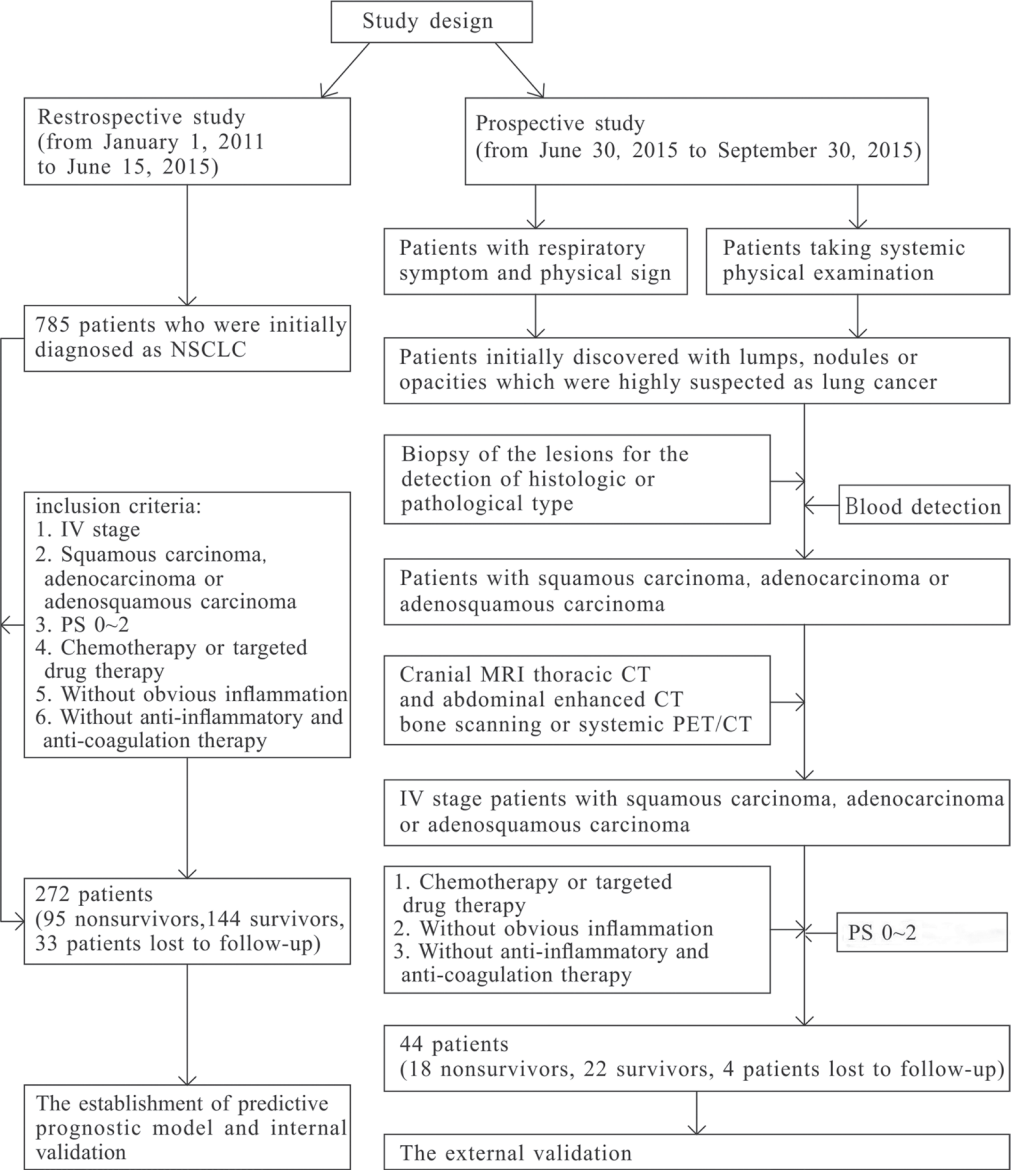
The study design

This was an open-label, single-center retrospective and prospective study. Its objectives were to establish a predictive prognostic model and calculate a novel hematological index that could serve as an independent prognostic factor. The whole research was consisted of a retrospective study and a prospective study (Figure 6). The former was applied to establish the predictive prognostic model and conduct the internal validation of the model, and the latter was used for external validation. The prospective study started on June 30, 2015 after the patients of the retrospective study were collected from January 1, 2011 to June 15, 2015. We defined 1-year overall survival as the primary endpoint of this study. The information of basic characteristics, hematological indexes, and image examination including cranial magnetic resonance imaging (MRI), thoracic computed tomography (CT), enhanced abdominal CT, or systemic positron emission tomography (PET)/CT taken before anti-tumor therapy was collected and analyzed. In the prospective study, 5–6 mL of peripheral venous blood of each enrolled patient was collected into an ethylenediaminetetraacetic acid (EDTA) tube after an 8-hour fast. The blood samples were transferred to a clinical laboratory within 30 minutes and analyzed.

The baseline characteristics collected for analysis included age (< 65 years vs. ≥ 65 years), gender (male vs. female), current smoking history (non-smokers vs. smokers), Performance Status score (PS; 0–1 vs. 2), lesion location (unilateral vs. bilateral), clinical TNM stage (IVa vs. IVb), M stage (1a vs. 1b vs. 1c), organ metastasis count (< 2 vs. ≥ 2), and pathological type (squamous vs. adenocarcinoma and adenosquamous carcinoma). Current smokers referred to patients with a smoking index of > 400 packs per year throughout their lives and having quit smoking less than 5 years prior. The eighth edition of the Union for International Cancer Control staging system was used to stage the lung tumors in all patients.

Four categories of hematological indexes were collected prior to the administration of any anti-cancer treatments. One referred to the inflammatory-immune reaction including the absolute value of white blood cells (WBC), neutrophils (N), and lymphocytes (L). Another one was routine hemostasis indexes such as platelets, fibrinogen (Fg), fibrin degradation product (FDP), and D-dimer (DD). Nutrition indexes containing red blood cell (RBC), HB, and albumin were collected as well. Several compound parameters including the NLR, neutrophil-(lymphocyte plus monocyte) ratio (NLMR), PLR, SII, platelet-D-dimer multiply (PDM), and prognostic nutritional index (PNI) were calculated. The above data were collectively known as inflammatory-immune-hemostasis-nutrition (IIHN) indexes in this study.

The patients’ follow-up data were collected by reviewing medical records and telephone interviews. All of the information was verified once again 15 days later.

### Patients

The study patients were classified into the retrospective study subgroup and the prospective study subgroup. The retrospective study population was collected from January 1, 2011 to June 15, 2015 according to strict inclusion and exclusion criteria. And the objects of prospective study were collected from 30 June, 2015 to 30 September, 2015. The inclusion criteria of the retrospective group are presented as the following aspects. First, the patients were initially diagnosed with stage IV NSCLC at the Department of Respiratory of Ruijin Hospital and had no moderate or severe inflammation. Second, the diagnosis of each patient with NSCLC was proven by histology or cellular pathology. Third, none of the patients had received anti-tumor, anti-inflammatory therapy, or anti-coagulation therapy prior to the blood sample examination. Fourth, all of the patients had an Eastern Cooperative Oncology Group Performance Status (PS) score of 0 to 2. Fifth, the patients received a definite intervention of chemotherapy and targeted drug therapy. Finally, Patients with a history of venous thrombosis, acute inflammatory disease, previous malignancy, or other severe systemic disease or for whom blood samples were not available were excluded from this study. Patients who did not undergo comprehensive imaging examinations including cranial MRI, thoracic CT, and abdominal enhanced CT or systemic PET/CT examination 1 month before or after the blood sample collection were excluded. In addition to the first five criteria that described above, the objects of the prospective study donot need to take all the examination. On the basis of the prognostic model established in the retrospective group, the laboratory results of neutrophil, lymphcyte, platelet, albumin and D-dimer are need, and the comprehensive imaging examinations listed as the retrospective group should be taken (Figure 6).

### Definition

In this study, NLMR, NLFDPA, NLFgA, and NLDA were new hematological indexes that were established using a combination of N, L, monocytes, FDP, Fg, D-dimer, and albumin, and the definitions of them were as follows: NLMR = N/(L + M), NLFDPA = N × FDP/(L × albumin), NLFgA = N × Fg/(L × albumin), and NLDA = N × D/(L × albumin). The optimal cut-points for NLFDPA, NLFgA, NLDA, and NLM were 0.54, 0.92, 0.15, and 2.3, respectively, and analyzed and calculated through the ROC curve. The definitions of other combined hematological indexes were as follows: NLR = N/L; PLR = P/L; PDM = P × D; SII = P × N/L; PNI = albumin + 5 × L. The references on the boundaries of NLR, PLR, and PNI were comparably consistent, the boundaries of NLR, PLR, and PNI were 3, 200, and 45, respectively [6, 7]. The cut-off values of the other indexes were calculated using the MedCalc software.

The normal reference ranges of the parameters in Ruijin Hospital are as follows: albumin, 35–55 g/L; WBC, 3.69–9.16 × 10^9^/L (female), 3.97–9.15 × 10^9^/L (male); N, 2–7 × 10^9^/L; L, 0.8–4 × 10^9^/L; RBC, 3.68–5.13 × 10^12^/L (female), 4.09–5.74 × 10^12^/L (male); HB, 113–151 g/L (female), 131–172 g/L (male); platelet (PLT), 101–320 × 10^9^/L, 85–303 × 10^9^/L (male); Fg, 1.8–3.5 g/L; FDP, 0–5 mg/L; and D-dimer, <0.55 mg/L. The abnormal groups were defined as RBC < LLN (lower limit of normal), HB < LLN, albumin <35 g/L, N >7 × 10^9^/L, L < 0.8 × 10^9^/L, NLR >3, NLMR >3.4, PLT > ULN (upper limit of normal), D-dimer >0.55 mg/L, Fg >3.5 g/L, FDP > 5 mg/L, PLR >200, PDM >272, SII ≥870, PNI < 45, NLFDPA > 0.54, NLFgA > 0.92, and NLDA > 0.15.

### Statistical analysis and graph creation

The clinical characteristics and hematological indexes of all the patients were presented as classified variables. The survival time of the stage IV NSCLC patients was described by median and interquartile range. The chi-square test was used to analyze the distribution of the death rate in different clinicopathological characteristics and hematological indexes, and to study the distribution of organ metastases in different group of NLDA. Univariate and multivariate Cox Regression analyses towards the retrospective study patients were used to establish the predictive prognostic model. Before takeing the analysis of Cox Regression, the linear correlation test was applied to investigate cox proportional hazards assumption. The variables significantly associated with the outcome in the univariate Cox Regression (two-sided *P* < 0.05) were included in the multivariate Cox Regression, and the interaction terms between those covariates were taken into consideration. The variables had been adjusted. All of the tests were two-sided, and *P* values < 0.05 were considered statistically significant. The above statistical analyses were performed using SPSS statistics version 17.0 for Windows.

Nomograms and calibration curves were created using R (version 2.1.0) to conduct the internal and external validation of the established predictive prognostic model, and the results were evaluated using the C-index (the larger the C-index, the more accurate the prognostic prediction). The nomogram is a widely used and pictorial method to predict the prognosis by using some valuable parameters. Using the nomogram, the prognosis of every patient could be accurately predicted. The C-index is an index that indicates the concordance level between the observed value and the value of expectation, and it is calculated to illustrate the predictive accuracy of the independent variables in the adjusted model. The accuracy of the predictive prognostic model were compared with that of TNM staging system (only *T* stage, *N* stage, and M stage). To determine the predictive values of the hematological indexes, the area under the curve (AUC) was calculated using the receiver operating characteristic (ROC) curve analysis. Other related parameters including sensitivity, specificity and the cut-off values of the novel combined hematological indexes were calculated using the MedCalc software.

Graphs were made using Graphpad Prism 5, R software, and SPSS 17.0.

## CONCLUSIONS

NLDA is significantly associated with organ metastases, and its level before anti-tumor therapy might be a convenient new economic parameter with higher specificity that can predict the prognosis of patients with clinical stage IV NSCLC in clinical practice in the future. Further large well-designed prospective multi-center studies should be conducted to confirm our findings. The mechanism behind the associations of elevated NLDA levels and poorer prognosis in patients with NSCLC needs further study.
